# Loss of Sensitivity to Rewards by Dopamine Neurons May Underlie Age-Related Increased Probability Discounting

**DOI:** 10.3389/fnagi.2020.00049

**Published:** 2020-03-06

**Authors:** Valerie L. Tryon, Phillip M. Baker, Jeffrey M. Long, Peter R. Rapp, Sheri J. Y. Mizumori

**Affiliations:** ^1^Department of Psychology, University of Washington, Seattle, WA, United States; ^2^Laboratory of Behavioral Neuroscience, Neurocognitive Aging Section, National Institute on Aging, National Institutes of Health, Baltimore, MD, United States; ^3^Program in Neuroscience, University of Washington, Seattle, WA, United States

**Keywords:** aging, probability discounting, ventral tegmentum, dopamine reward responses, memory

## Abstract

Normative aging is known to affect how decisions are made in risky situations. Although important individual variability exists, on average, aging is accompanied by greater risk aversion. Here the behavioral and neural mechanisms of greater risk aversion were examined in young and old rats trained on an instrumental probability discounting task. Consistent with the literature, old rats showed greater discounting of reward value when the probability of obtaining rewards dropped below 100%. Behaviorally, reward magnitude discrimination was the same between young and old rats, and yet these same rats exhibited reduced sensitivity to positive, but not negative, choice outcomes. The latter behavioral result was congruent with additional findings that the aged ventral tegmental neurons (including dopamine cells) were less responsive to rewards when compared to the same cell types recorded from young animals. In sum, it appears that reduced responses of dopamine neurons to rewards contribute to aging-related changes in risky decisions.

## Introduction

According to the world health organization (WHO), in almost every country, the proportion of people aged over 60 years is growing faster than any other age group as a result of both longer life expectancy and declining fertility rates. An accompaniment of aging is, changes in behavior, that is governed by underlying changes in brain physiology. Understanding how the brain normally ages and affects behavior will allow us to adapt to the new population landscape. Normal cognitive aging does not usually result in great losses in the number of neurons but is often accompanied by changes in decision making and memory functions even in the absence of neurodegenerative disease (Rapp and Gallagher, 1996; Morrison and Hof, 1997; Rapp et al., 2002; Gallagher et al., 2003; Rosenzweig and Barnes, 2003; Fletcher and Rapp, 2012; Orsini et al., 2019). Thus, an important avenue of current research is to understand how behavior changes with age, and the mechanisms mediating normative brain aging. While studies exploiting the power of *in vivo* neuroimaging in humans have yielded important insights, the approaches available in animal models are necessary for a comprehensive cell biological account of the neural basis of cognitive aging. The latter is especially relevant since similar behavioral and cognitive patterns of change are observed in old rodents and primates (Gallagher et al., 2011).

Neurocognitive processes that are mediated by the hippocampus, such as declarative memory and pattern separation, or by the dorsolateral prefrontal cortex (PFC), such as cognitive flexibility and working memory, are among the most vulnerable in aging (Barense et al., 2002; Dumitriu et al., 2010; Morrison and Baxter, 2012). It is thought that decline in separate neural systems occurs independently from each other; thus, declines in PFC function do not necessarily imply deficits in hippocampal function and vice versa (Moore et al., 2009; Gallagher et al., 2011; Fletcher and Rapp, 2012). We also know that cost-benefit decision making is altered with age, albeit to what extent is less clear. In humans, older individuals tend to choose sure gains and avoid sure losses (Mather et al., 2012), avoid risky choices (Lee et al., 2008), and gains and losses are encoded asymmetrically (Samanez-Larkin et al., 2007). However, aging has also been associated with an increase in making risky choices in some rats and humans, and risk-aversion in others (Mata et al., 2011; Gilbert et al., 2012; Samson et al., 2015). The findings of individual differences in probability discounting in aged rats are consistent with evidence for individual differences in aged rat, monkey, and human performance in other cognitive domains (Gallagher et al., 1993, 2011; Barense et al., 2002; Schoenbaum et al., 2006; Bizon et al., 2009; Morrison and Baxter, 2012). Probability discounting is the change in the subjective value of a reward based on the probability of receiving it, e.g., a reward has reduced value and is “discounted” if the probability of receiving it is low. On average, however, older individuals (animals and humans) tend to display more risk-aversion during risk-based decision making. Therefore, the goal of the current study was to investigate in more detail how normal aging affects risk-based decision making. We selected a rodent model that features increased individual differences in memory with aging, providing a setting for asking whether these effects are coupled with changes in decision making, potentially pointing to shared underlying substrates. Our probability discounting task tested rats’ preference for a lever that led to a large reward with varying probability (the risky option) or a lever that lead to a small reward 100% of the time (the certain option). The probabilities associated with the risky lever were 100%, 50%, 25%, and 12.5%. The central question was whether aged rats show a preference for the small, certain reward over the risky one, even when the outcome would be better if the risky option was selected. Like aged humans tested on similar tasks, we expected aged rats to display significantly reduced choice of the large risky reward, or increased discounting behavior, compared to young controls.

Also of interest was the question of how aging may influence risk-based decision making because we know that normal aging does not affect all brain systems equally as some systems are more prone to functional decline over time. For example, dopamine function declines across the life-span, and this decline has been correlated with age-related cognitive deficits (Bäckman et al., 2006, 2010; Rollo, 2009). Midbrain dopamine (DA) neurons of the ventral tegmental area (VTA) appear highly evolved to have strong responses to rewards and biologically salient events. They exhibit burst-like activity in response to primary rewards such as food and water (Wise, 2004; Fields et al., 2007). It is thought that these specialized responses to salient and rewarding events are important for the learning and selection of appropriate behavioral responses. Therefore, DA functional decline over the lifespan could alter cognition reliant on DA. Indeed, evidence suggests that an age-related decrease in DA release is linked to poorer working memory function and perceptual speed (Bäckman et al., 2010). Additionally, DA hypofunction has been implicated in decreased sensitivity to changes in reward magnitudes (Dreher et al., 2008). However, it is not known if the functional decline in DA function is linked to altered risk-based decision making observed in old age. We know that midbrain DA neurons encode the uncertainty of probabilistic rewards (Fiorillo et al., 2003) and that alteration of DA signaling alters risky choice (St. Onge and Floresco, 2009; St. Onge et al., 2010). Therefore, another goal of the current project was to investigate how reward processing may change in the aged brain by recording neural activity from DA neurons of the VTA.

## Materials and Methods

### Subjects

Fifteen 6–9 months and 19 25–26-month-old male Long-Evans rats were received from the Laboratory of Behavioral Neuroscience at the National Institute on Aging, National Institutes of Health (Baltimore, MD, USA). All rats are originally from the Charles River facility (Raleigh, NC, USA). Twenty-eight rats (12 young and 16 aged) were used in the behavioral experiments and six rats (three young and three aged) were used for the DA recording experiment. Upon arrival, rats were housed individually in Plexiglas cages and were maintained on a 12 h light/dark cycle (lights on at 7:00 AM). All behavioral experiments were performed during the light phase of the cycle. Each rat was allowed access to water ad libitum and food-deprived to 80–85% of its ad libitum feeding weight. In some cases of extreme obesity, rats were further restricted to 75% of their free-feeding weight with oversight from the veterinary staff. Rats were handled and weighed daily. Rats that developed any health concerns were evaluated by veterinary staff and excluded from the experiment immediately if appropriate. All animal care and use were conducted in accordance with the University of Washington’s and National Institute of Aging’s Institutional Animal Care and Use Committee guidelines.

### Behavioral Testing Procedures

#### Water Maze Testing

Prior to arrival at the University of Washington (UW), standard water maze training (for detailed description, see Gallagher et al., 1993) was conducted for all rats. Briefly, training took place across 8 consecutive days, three trials per day (each using a 90 s cutoff), with a 60-s intertrial interval. Every sixth trial was a probe test in which the escape platform was initially retracted to the bottom of the maze for 30 s and then made accessible to permit escape. The performance was evaluated according to learning index scores (LISs), as described previously (Gallagher et al., 1993). Briefly, the LISs were calculated as the weighted average proximity (in centimeters) to the hidden escape location across probe trials. This measure is optimized for identifying reliable individual differences in memory and was used to classify aged animals as either aged unimpaired (AU), or aged impaired (AI), using criteria validated in earlier research (Gallagher et al., 1993). By this measure, lower values reflect closer proximity to the escape location and better spatial learning. The day after completing the spatial protocol, rats were tested on a one-session, hippocampus-independent cued version of the Morris water maze. Six trials were given from multiple start locations, 60-s maximum trial length. No animals that failed cue training were included in the present experiment.

#### Probability-Discounting Task

##### Apparatus

All UW behavioral testing and neural recordings took place in Med PC operant chambers (30.5 × 24 × 21 cm; Med-Associates, St. Albans, VT, USA). Operant chambers were enclosed in sound-attenuating boxes with a fan to provide ventilation. While standard chambers are equipped with a metal grid floor, custom cut Plexiglas covered the floors for all rats to provide aged animals with a comfortable standing surface. The chamber was illuminated by a houselight on one side of the chamber. On the opposite wall from the houselight was a food cup in the center of one wall and two retractable metal levers on either side of the food cup. A single light was located above each lever. For recording experiments, the food receptacle was custom built to extend from the wall to allow implanted animals to easily obtain the food reward. Additionally, the metal levers were coated in plastic to dampen electrical noise during neural recording experiments. Food rewards were 45 mg sugar pellets (Bioserv) that were dispensed from a pellet dispenser. Nose-pokes, or when the animals’ nose-first approached the food cup, were recorded *via* two infrared photo beams on either side of the food cup.

##### Pre-training

Animals underwent identical pre-training procedures for both experiments. Training protocols were adapted from St. Onge and Floresco (2009). Once food restricted to 80–85% of their free-feeding weight, rats were given sugar pellets in their home cage to avoid neophobia for 1–2 days prior to training in the operant chamber. Then, rats were randomly assigned to one of the operant chambers that would then stay consistent throughout the training. The first day of pretraining consisted of lever-press training at a fixed-ratio 1 schedule. Crushed sugar pellets were placed on the lever to encourage the rat to press the lever until the rat started pressing reliably. Sugar pellets were also placed in the food cup. Rats were trained on the FR1 schedule of lever pressing for a single lever until they reached the criteria of 60 lever presses within 30 min after which they switched to the same training protocol on the other lever. The first lever presentation was counterbalanced between rats. Then, rats trained on a simplified version of the probability discounting task. This task consisted of 90 trials during which one of the two levers was extended with the house light illuminated (left and the right lever was presented once for every two trials and the order of presentation was randomized within a pair of trials). A lever press during one of these trials resulted in the delivery of a sugar pellet with a 50% probability. Once pressed, the lever retracted, the house light turned off and another trial was initiated after an inter-trial interval. If the rat failed to press the lever within 10 s of lever presentation, the lever retracted, the house light turned off, and the trial was recorded as an omission. Every trial was 40 s long regardless of the behavior of the animal. This procedure was used to allow the rats to learn about the probabilistic nature of lever pressing. Rats were trained on this version of the task until they reached criterion, defined as an omission of 10 or fewer trials per session. Days to reach criterion for each task varied from rat to rat (see “Results” section).

##### Probability-Discounting Task: Behavioral Training

The task was based on previous studies investigating probability discounting (Cardinal and Howes, 2005; St. Onge and Floresco, 2009). After pretraining, rats performed 48-min sessions consisting of 72 trials 5 days a week. Each session was separated into four different probability blocks of 18 trials. A trial began every 40 s with the illumination of the houselight and, 3 s later, insertion of one or both levers into the chamber. One lever was designated the Large/Risky lever, the other the Small/Certain lever, which remained consistent throughout training (counterbalanced left/right). Half of the rats were assigned their preferred lever as the risky lever and the other half were assigned their non-preferred lever as risky. If the rat did not respond by pressing a lever within 10 s of lever presentation, the lights turned off, the levers were retracted, and the trial was scored as an omission. When a lever was chosen, both levers retracted. Pressing a certain lever led to the delivery of one sugar pellet 100% of the time. Pressing the “risky” lever led to four sugar pellets with varying probability across trial blocks. The probability blocks were presented in descending order. The first block was associated with a 100% probability of receiving the large reward when the risky lever was pressed, then 50%, then 25%, and finally 12.5%. When food was delivered, the houselight remained on for another 4 s after a response was made, after which the chamber reverted to the intertrial state (darkness). Multiple pellets were delivered 0.5 s apart. The four probability blocks were separated into eight forced-choice trials where only one lever was presented (four trials for each lever, randomized in pairs) allowing rats to learn the amount of food associated with each lever press and the respective probability of receiving reinforcement over each block. This was followed by 10 free-choice trials, where both levers were presented and the animal chose either the Small/Certain or the Large/Risky lever. For each session and trial block, the probability of receiving the risky reward was drawn from a set probability distribution. Therefore, on any given day, the probabilities in each block may vary, but on average across many training days, the actual probability experienced by the rat will approximate the set value. Using these probabilities, selection of the Large/Risky lever would be advantageous in the first two blocks, and disadvantageous in the last block, whereas rats could obtain an equivalent number of food pellets after responding on either lever during the 25% block. Therefore, in the last three trial blocks of this task, the selection of the larger reward option carried with it an inherent “risk” of not obtaining any reward on a given trial.

##### Probability-Discounting During Neural Recording

A modified version of the probability discounting task was used for rats in the recording experiment to increase the number of like-choice trials (20 for each probability instead of 10) and ensure participation in the lower probability blocks, particularly for the aged rats. Rats were trained on a probability discounting task until behavioral criterion was reached using only two probability blocks: 80% and 20%. Each probability block consisted of 10 forced-choice trials and 20 free-choice trials. The behavioral criterion was defined as stable behavior over 5 days (two-way ANOVA: no effect for day, a significant effect of probability).

### Neural Recording Procedures

#### Electrode Preparation and Surgical Procedures

Recording tetrodes were constructed from 20 μm lacquer-coated tungsten wires (California Fine Wire) and mounted on one of two independently adjustable custom-built microdrives (three tetrodes per microdrive). Tetrode tips were gold-plated to reduce impedances to 0.1–0.4 MΩ (tested at 1 kHz). To implant recording electrodes, each rat was placed in an induction chamber and deeply anesthetized under isoflurane (4% mix with oxygen at a flow rate of 1 L/min). Under deep anesthesia, the animal was placed in a stereotaxic instrument (David Kopf Instruments, Tujunga, CA, USA) and anesthesia was maintained throughout surgery by isoflurane (1–2.5%) delivered *via* a nosecone. The skull was exposed and adjusted to place bregma and lambda on the same horizontal plane. After small burr holes were drilled, the microdrives were unilaterally implanted into the VTA (Young: −6.0 mm posterior, 0.5 mm lateral, and 7.0 mm ventral to Bregma. Aged: −7.5 mm posterior, 0.5 mm lateral, and 7.0 mm ventral to Bregma). An additional set of aged rats underwent a lesioning procedure to obtain the optimal stereotaxic coordinates for the VTA of young and aged rats since initial surgeries were unsuccessful in targeting the VTA of aged rats using standard atlas coordinates. Microdrive arrays were secured in place with anchoring screws and dental cement. Rats recovered for 7 days, during which they were weighed and handled daily.

#### Single-Unit Recording and Postsurgical Procedures

After a week of recovery, rats were returned to a food-restricted diet and spontaneous neural activity in the VTA was monitored as follows: the electronic interface board (Neuralynx, Bozeman, MT, USA) of the microdrives were connected to preamplifiers, and the outputs were transferred to a Cheetah data acquisition system (Neuralynx). Signals were filtered between 0.6 and 6 kHz and digitized at 16 kHz. Acquired waveforms were not inverted. Neuronal spikes were recorded for 2 ms after the voltage deflection exceeded a predetermined threshold at 500–7000X amplification. If no units were encountered, tetrodes were lowered in 40 μm increments to target new units. A non-motorized commutator (SwivElectra, Crist Instrument Company, Hagerstown, MD, USA) with custom Neuralynx adapters was mounted above the operant chamber and used to prevent the tether from getting tangled. A video camera mounted on the ceiling of the chamber recorded video, and data were relayed to the acquisition system. Once clearly isolated and stable units were found, the probability discounting task and recording began. Daily recording sessions included behavioral data and simultaneously recorded neural data that were time-stamped in the form of TTL pulses instantaneously sent to the neural recording system *via* the SuperPort TTL card (DIG-726) and the Cheetah data acquisition system. The commands sent by Med PC to run the behavioral experiment were also recorded in the Neuralynx event file as TTL pulses *via* a SmartCtrl connection panel (SG-716B; Zheng and Ycu, 2012). After each completed session, tetrodes were advanced about 40 μm. This resulted in the appearance of stable records from new cells the following day. Experimental sessions continued until tetrodes passed through the VTA based on the distance traveled from the brain surface.

#### Neural Data Analysis

Single units were isolated using an Offline Sorter (Plexon). Various waveform features, such as the relative peak, valley, width, and principle component, were compared across multiple units simultaneously recorded from the four wires of a tetrode. Only units showing good recording stability across the entire recording session were included. Further analysis of the sorted units was performed with custom Matlab software (Mathworks). To examine the reward-related responses of histologically verified VTA neurons, peri-event time histograms (PETHs) were constructed at 4.0 s around the time of all reward acquisition-triggered events. A bin size of 100 ms was used for all PETHs. VTA cells were identified based on phasic responses to reward acquisition, lever cues, and pellet cues. A VTA neuron was considered a reward or cue responsive if it at least doubled its firing rate during the 300 ms from cue or reward acquisition onset compared to its baseline firing rate. Putative DA and non-DA cells were classified using a cluster analysis developed to identify DA cells in the rodent VTA (Roesch et al., 2007; Jin and Costa, 2010; Jo et al., 2013). Detailed information on this analysis can be found in previous reports (Roesch et al., 2007; Takahashi et al., 2009, 2011; Jo et al., 2013). Briefly, using the tetrode channel with the largest peak-to-valley amplitude, two basic characteristics of the average spike waveform were determined for each cell: (1) the half time of the spike duration (i.e., measured between the first valley and the next peak); and (2) the amplitude ratio of the first positive peak and negative valley in a waveform [(*n* − *p*)/(*n* + *p*), with *n* as the first negative valley and p as the first positive peak]. A scatter plot including all VTA cells was then constructed for young and aged rats together, as well as for each age group separately. The cluster that included neurons with waveforms showing a broad half duration and low amplitude ratio was putatively classified as DAergic. Neurons that fell into multiple clusters were not classified. Spontaneous firing properties of putative DA cells were calculated from data collected during the ITIs (i.e., when rats were not engaged in the task-related behavior).

#### Histology

After the completion of all recording sessions, tetrode locations were verified. Rats were deeply anesthetized under 4% isoflurane. The final position of each tetrode was marked by passing a 15A current through a subset of the tetrode tips for 15 s Then, the animals were given an overdose of sodium pentobarbital and transcardially perfused with 0.9% saline and a 10% formaldehyde solution. Brains were stored in a 10% formalin–30% sucrose solution at 4°C for 72 h. The brains were frozen and then cut in coronal sections (45 μm) on a freezing sliding microtome. The sections were then mounted on gelatin-coated slides, stained with cresyl violet, and examined under light microscopy. Only cells verified to be recorded in VTA were included in the data analysis.

### Statistical Analyses

Statistical analyses were performed using SPSS 22.0, Graphpad Prism 6.0, or custom Matlab software (MathWorks, Natick, MA, USA). The statistical tests used are indicated in the “Results” section where appropriate.

## Results

### Age Effects on Probability Discounting Behavior

**Figure 1:**
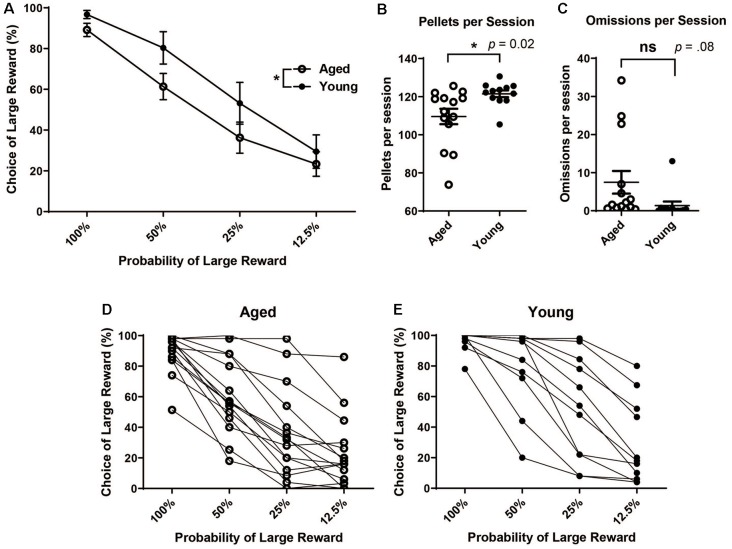
**(A)** Mean (± SEM) choice of large rewards for all probability blocks grouped by age. Scores reflect rats’ choice of the large reward lever during the choice trials in which rats participated (i.e., not including omission trials). Aged rats chose the large risky lever significantly less often than young rats (*F*_(1,104)_ = 7.93, *p* < 0.01). **(B)** The Mean number of pellets received per session by age group. Young rats received more pellets per session on average than aged rats (*t*_(24)_ = 2.54, *p* = 0.02). **(C)** Mean number of omitted trials per session. There was not a significant difference in the number of omitted trials between groups (*t*_(24)_ = 1.82, *p* = 0.08). **(D)** Individual aged rats’ choice behavior across all four probability blocks. **(E)** Individual young rats’ choice behavior across all four probability blocks. Open circles: aged rats, *n* = 16; Closed circles: young rats, *n* = 12.

After stable behavior was reached, the mean choice of the large reward at each probability block was calculated for each rat. Each rat’s performance was averaged over the five-day stable behavior period. Performance scores reflect rats’ choice of the large reward lever during the choice blocks. The choice of the large reward was calculated only from those trials in which rats participated (i.e., and not omission trials). Then, rats’ scores were grouped by age. A mixed factorial ANOVA was run to assess the effects of age and probability on choice behavior. There was a significant main effect of probability (*F*_(3,104)_ = 43.53, *p* < 0.001) and a significant main effect of age (*F*_(1,104)_ = 7.93, *p* < 0.01), with aged rats showing reduced choice of the large reward over the four probability blocks (Figure 1A). The choice of the large/risky reward was not significantly different between aged and young rats when outcomes were certain, i.e., when the probability of the “risky” reward was 100% (independent *t*-test, *t*_(25)_ = 1.84, *p* = 0.08). Instead, differences arose when risk was introduced, even when it was more beneficial to continue choosing the risky option, i.e., during the 50% block (independent *t*-test, *t*_(25)_ = 1.99, *p* = 0.02) but not choices were equal, i.e during the 25% block (independent *t*-test, *t*_(25)_ = 1.48, *p* = 0.08). Based on the probability of receiving a reward, optimal behavior would be 100% choice of the large reward, risky lever during the 100% and 50% block, equal during the 25%, and 100% choice of the small reward certain lever during the 12.5% block. There was no difference between the groups’ behavior for 100% or 12.5% blocks. However, the young rats had more optimal behavior as they chose the large reward risky lever more frequently, as a group, during the 50% block. Additionally, young rats received more sugar pellets (reward) per session than aged rats (Figure 1B; unpaired *t*-test, *t*_(24)_ = 2.54, *p* = 0.02). This difference in the amount of reward received was not due to more omissions of choice trials by aged rats (Figure 1C; unpaired *t*-test, *t*_(24)_ = 1.82, *p* = 0.08). When viewing each rat’s discounting behavior individually (Figures 1D,E), the trend for aged animals to choose the large risky reward less often than their younger counterparts become more clear, albeit there is a certain amount of individual variability in the level of discounting within both age groups.

**Figure 2:**
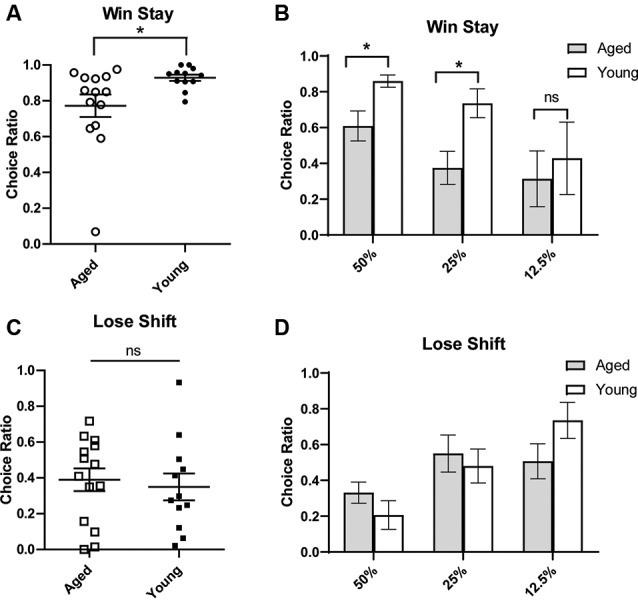
Win-stay **(A,B)** and lose-shift choice ratios **(C,D)**. A win is defined as a choice for the large/risky lever that resulted in a reward. (Top) The choice ratio for win-stay represents the ratio of choices of the risky (as opposed to safe) lever following a “win” risky trial. **(A)** Individual rat win-stay choice ratios for aged and young groups. Aged rats showed significantly reduced win-stay ratios compared to young rats (independent *t*-test, *t*_(24)_ = 2.42, *p* = 0.01). **(B)** This effect was specifically driven by a decrease in the choice ratio in the 50% and 25% blocks (*p’s* < 0.05). (Bottom) A loss is defined as a choice for the large/risky lever that did not yield a reward. The choice ratio for lose-shift represents the ratio of choices for the small/certain lever after a “loss.” **(C)** Individual rat lose-shift choice ratios for aged and young animals. There was no difference in lose-shift behavior by age group (independent *t*-test, *t*_(24)_ = 0.69, *p* = 0.25). **(D)** There was no difference in the lose-shift choice ratio in 50% and 25% blocks. Open symbols: aged rats, *n* = 16; Closed symbols: young rats, *n* = 12. *p < 0.05; ns, not significant, *p* > 0.05.

To gain more insight into how aged rats’ choices led to increased discounting, we next conducted a win-stay, lose-shift analysis. The ability to make flexible decisions based on choice feedback is important for optimal decision making. However, being too sensitive to losses or not sensitive enough to positive feedback may reduce gains in probabilistic paradigms. Aged rats have previously been shown to have win-stay and lose-shift deficits (Means and Holsten, 1992). A win-stay deficit is defined as not repeating a choice that was previously rewarded, even when it is beneficial to do so. A lose-shift deficit is defined as not switching to the other option after a choice was not rewarded/correct. Win-stay and lose-shift behavior are thought to reflect sensitivity to positive and negative reinforcement, respectively. Thus, we wanted to assess if the increase in probability discounting observed with age-related to distinct changes in sensitivity to positive or negative reinforcement. To do this, a win-stay and lose-shift choice ratio were calculated for each rat. For win-stay, the ratio reflected the number of choices for the risky lever after a risky win out of the total number of trials for which there was a risky gain. The ratio of lose-shift choices was calculated as the number of choices for the small certain lever after the risky lever was chosen and no reward was delivered, i.e., a loss, out of the total number of loss trials. These ratios were calculated for each rat, and rats were then grouped by age for analysis. It was found that aged rats had significantly reduced win-stay choice ratios compared to young rats (independent *t*-test, *t*_(24)_ = 2.42, *p* = 0.02; Figures 2A,B). We examined if this effect was being driven primarily by the 50% and 25% blocks and found that aged rats had significantly reduced win-stay choice ratios in these blocks (two-way ANOVA, significant main effect of age *p* < 0.01; Bonferroni *post hoc* comparisons 50%: *p* < 0.05; 25%: *p* < 0.01; Figure 2B). Win-stay choice ratios were not significantly different during the 12.5% block (*p* > 0.05; Figure 2B). However, when lose-shift choices were compared, aged and young rats displayed similar lose-shift behavior collapsed across all probabilities (independent *t*-test, *t*_(24)_ = 0.69, *p* = 0.25; Figure 2C), as well when examining each probability separately (Figure 2D; two-way ANOVA, *F*_(1,68)_ = 0.02; *p* = 0.89). This suggests that aged rats have a selective deficit in maintaining risky choices after positive reinforcement and are not necessarily more loss-averse than their younger counterparts.

**Figure 3:**
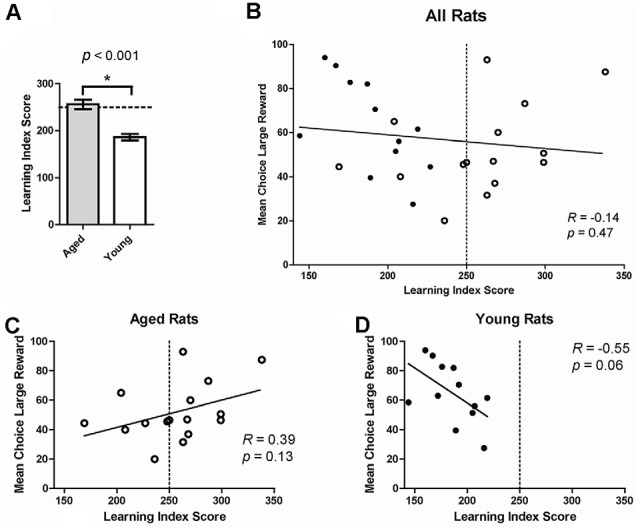
**(A)** Mean learning index score (LIS) by age group. Aged rats had significantly higher LISs (indicating poorer spatial memory) compared to young rats, even when collapsing aged impaired and unimpaired groups (one-tailed independent *t*-test, *t*_(26)_ = 5.23, *p* < 0.0001). **(B)** The Mean choice of large reward collapsed across all probability blocks on the probability discounting task (%, Y-axis) correlated to LIS on the water maze (X-axis). No significant relationship was observed between choice behavior on the probability discounting task and water maze performance (Pearson correlation, *R* = −0.14, *p* = 0.47). The dotted line represents the threshold after which (to the right) rats are considered impaired on the water maze. Open circles: aged rats, *n* = 16; Closed circles: young rats, *n* = 12. **(C)** Relationship between risky choice and water maze performance for aged rats. No statistically significant relationship was found between these two variables (Pearson correlation, *R* = 0.39, *p* = 0.13). **(D)** Relationship between risky choice and water maze performance for young rats. No significant relationship was found between these two variables (Pearson correlation, *R* = −0.55, *p* = 0.06) although a trend is suggested. *p < 0.05.

We next assessed if age-related changes on risk-based decision making were related to deficits in spatial memory. Previous research suggests that deficits in spatial memory tasks with age can be selective, indicating that age-related decline in the hippocampus happens independently of changes in other brain regions (Gilbert et al., 2012; Samson et al., 2015). To assess this in the current study, individual choice behavior on the probability discounting task was correlated with performance on the water maze. A LIS was calculated based on performance on multiple probe trials on the water maze. LIS reflects the average proximity of the animal during probe trials to the training location of the escape platform; lower index scores indicate more accurate proximity and is used as an approximation of spatial memory (Gallagher et al., 1993). Rats are considered “impaired” with an LIS ≥250. LISs have been shown to be associated with age-related changes in spatial memory (Gallagher et al., 1993; Nicolle et al., 1999). As expected, aged rats had significantly higher LISs compared to young rats, even when collapsing aged impaired and unimpaired groups (one-tailed independent *t*-test, *t*_(26)_ = 5.23, *p* < 0.0001; Figure 3A). When grouping all 28 young and old rats together, no significant relationship between choice behavior on the probability discounting task and water maze performance was observed (Pearson correlation, *R* = -0.14, *p* = 0.47; Figure 3B). We then compared choice behavior and water maze performance separately by age group. While not statistically significant, there was a positive relationship between large reward choice and LIS in aged rats (*R* = 0.39, *p* = 0.13; Figure 3C), and a negative relationship between large reward choice behavior and LIS in young rats (*R* = -0.55, *p* = 0.06; Figure 3D). There was also a significant difference between the two correlations (Fisher’s *z* = 2.26; *p* = 0.02), showing the relationship between risky reward choice and spatial memory performance was different for the two age groups. In other words, the more an aged rat chose the risky reward the more spatially impaired they were on the water maze task, while young rats displayed the inverse relationship between the two variables.

### Age Effects on Neural Responses During Probability Discounting

**Figure 4:**
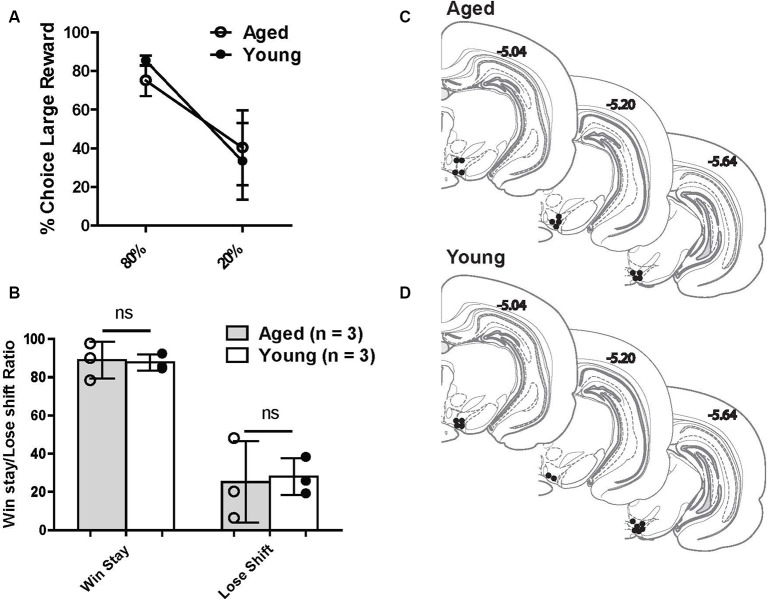
**(A)** Risk-based decisions by age group for recording experiment: mean (±SEM) choice of large rewards for 80% and 20% probability blocks grouped by age. Scores reflect rats’ choice of the large reward lever during the choice trials. There was no significant difference in choice behavior between age groups. **(B)** Win-stay and lose-shift choice behavior for aged and young rats. Open circles: aged rats, *n* = 3; Closed circles: young rats, *n* = 3. **(C,D)** Histological reconstruction of terminal tetrode tip locations in aged and young rats. ns = not significant, *p* > 0.05.

Another aim of the current study was to investigate VTA (both DA and non-DA neurons) neural responses to probabilistic rewards in aged and young animals. Loss of the appropriate phasic response to rewards may lead aged rats to interpret the risky reward as less reinforcing and thus be less sensitive to positive reinforcement. This could be a possible mechanism that reduces the ratio of win-stay choice behavior in aged rats. We recorded 269 neurons from the VTA in six rats (Figures 4C,D) while rats performed a two-probability version of the task. Aged and young rats did not have significantly different choice behavior in this version of the task (Figures 4A,B).

**Figure 5:**
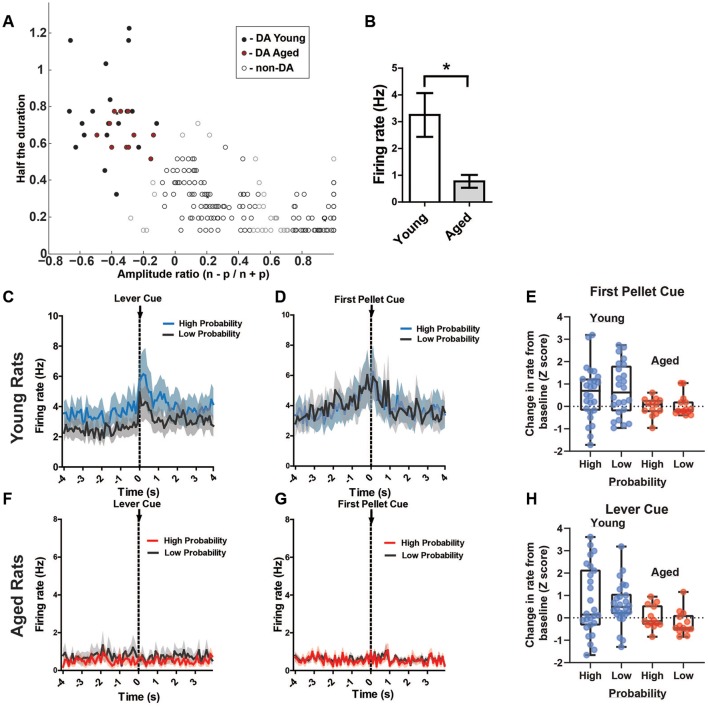
**(A)** Results of cluster analysis for all ventral tegmental area (VTA) neurons (*N* = 269). Putative dopamine (DA) cells were identified based on half spike duration and the amplitude ratio of the initial negative valley (*n*) and positive peak (*p*). Closed circles are putative DA neurons (*n* = 38); open circles are classified as non-DA neurons (*n* = 231). **(B)** The overall rate of the putative aged DA neurons (0.77 ± 0.24 spikes/s) was significantly reduced compared to young rats (3.25 ± 0.82 spikes/s; *t*_(35)_ = 2.24, *p* = 0.02). **(C)** Mean firing rate (Hz) of young rats’ putative DA neurons (*n* = 26) centered around the lever cue (±4.0 s). Blue traces indicate responses during the 80% block; gray traces represent the 20% block. DA neurons’ responses to the lever cue was significantly greater than baseline firing rates (repeated measures ANOVA, significant main effect of time, *F*_(2,25)_ = 10.92, *p* < 0.001) and this response was greater during the high probability block (Bonferroni *post hoc* multiple comparisons test, *t* = 2.32, *p* = 0.01). **(D)** Mean firing rate (Hz) of young rats’ putative DA neurons centered around the first pellet cue (±4.0 s). The neurons significantly increased firing rate to the pellet cue compared to baseline (repeated measures ANOVA, significant main effect of time, *F*_(2,25)_ = 10.49, *p* = 0.01), but they did not show significantly increased firing rates to this cue during the 80% block compared to the 20% block (Bonferroni *post hoc* multiple comparisons tests, *t* = 0.77, *p* > 0.05). **(F)** Mean rate (Hz) of aged rats’ putative DA neurons (*n* = 11) centered around the lever cue (±4.0 s). Red traces indicate responses during the 80% block; gray traces represent the 20% block. **(G)** Mean rate (Hz) of aged rats’ putative DA neurons centered around the first pellet cue (±4.0 s). **(E)** Individual DA neurons’ normalized firing rate change from baseline to the first pellet cue (*Z* scores). **(H)** Individual DA neurons’ normalized firing rate change from baseline to the lever cue (*Z* scores). Blue dots represent young rats’ neurons; red dots represent aged rats’ neurons. These changes in firing rate are separated by the high and low probability blocks. *p < 0.05.

Of those 269 recorded neurons, 38 (14.1%) were identified as dopamine neurons based on previously established electrophysiological criteria (see “Materials and Methods” section; Roesch et al., 2007; Jo et al., 2013; Figure 5A). Putative DA neuron numbers were similar to previous reports (Roesch et al., 2007; 36/258; 13.9% and Jo et al., 2013; 203/905; 22%). However, there were proportionally fewer DA neurons recorded from aged rats than young [Aged: 11 DA neurons out of 115 total (10.4%); Young: 26 DA neurons out of 154 total (16.9%)]. However, this proportional frequency was not significantly different between groups (*χ*^2^ = 2.97, *p* = 0.08). Aged rats’ DA neurons had a significantly lower firing rate than young rats’ DA neurons (*p* < 0.05; Figure 5B). To potentially account for this age-difference, it is important to note that DA neurons show functional changes with age. While less sensitive to neuronal loss than the nigrostriatal system, DA neurons of the VTA display functional decline as DA metabolites (DOPAC, HVA) decline with age in the rat VTA (Goudsmit et al., 1990). VTA DA neurons display axonal degeneration, a build-up of amyloid precursor protein and alpha-synuclein, and loss of tyrosine hydroxylase (TH) despite no obvious neurodegeneration (Cruz-Muros et al., 2007). Most importantly, age can affect the electrophysiological properties of recorded DA neurons. In a previous report, it was found that DA neurons from aged mouse substantia nigra showed lower spontaneous firing rates, impaired firing fidelity, narrower spike widths, and decreased L-type calcium currents (Branch et al., 2014). Therefore, using previously established methods to identify putative young adult DA neurons in the VTA may lead to exclusion of DA neurons in the aged rat. Due to the above-described age changes in DA neural integrity, we initially analyzed DA cell responses after identifying cells as DAergic based on published criteria (e.g., Jo et al., 2013; Figure 5A). Then, we performed the same analysis on all VTA cell records from young and old rats.

Of interest were DA neuron responses to probabilistic cues that indicate the availability of reward (lever cue), cues indicating when a choice resulted in a reward (pellet cue), and the receipt of the reward itself (first nose poke after a reward was delivered). Based on previous research, it is known that DA neurons encode the value of probabilistic rewards: neurons exhibit a lower firing rate to cues that are associated with a lower probability of receiving reward compared to cues that are associated with a high probability of receiving reward, and DA neurons respond more to a primary reward that was preceded by a cue associated with a low probability of receipt (Fiorillo et al., 2003). Therefore, we hypothesized that DA neurons would exhibit phasic responses to the lever cue, with a higher firing rate to the cue during the 80% block compared to the 20% block. Additionally, we expected higher phasic firing of DA neurons to the pellet cue, with a higher firing rate during the 20% block compared to the 80% block. In the young rats, DA neurons indeed exhibited phasic firing to the lever cue that was significantly greater than baseline firing rates (repeated measures ANOVA, significant main effect of time, *F*_(2,25)_ = 10.92, *p* < 0.001; Figure 5C) and this phasic response was greater during the high probability block (Bonferroni *post hoc* multiple comparisons tests, *t* = 2.32, *p* = 0.01). When examining these same neurons’ responses to the pellet cue, we found that they exhibited significantly increased firing rate to the pellet cue compared to baseline (repeated measures ANOVA, significant main effect of time, *F*_(2,25)_ = 10.49, *p* = 0.01; Figure 5D), but they did not show significantly increased firing rate to this cue during the 80% block compared to the 20% block (Bonferroni *post hoc* multiple comparisons tests, *t* = 0.77, *p* > 0.05). While there was a significant increase in firing rate to the first pellet cue and lever cue in the majority of young rats’ DA, we did find great variability in the magnitude and direction of change when examining the neurons individually (Figures 5G,H). The aged rats’ neurons, by comparison, did not show any detectable responses to any of these cues as a group (Figures 5E,F). Additionally, overall rate of the putative aged DA neurons (0.77 ± 0.24 spikes/s) was significantly reduced compared to young rats (3.25 ± 0.82 spikes/s; *t*_(35)_ = 2.24, *p* = 0.02). Compared to young rats, individual DA neurons from aged rats showed a consistent pattern of a lower magnitude of change in firing rate to these cues (Figures 5G,H).

**Figure 6:**
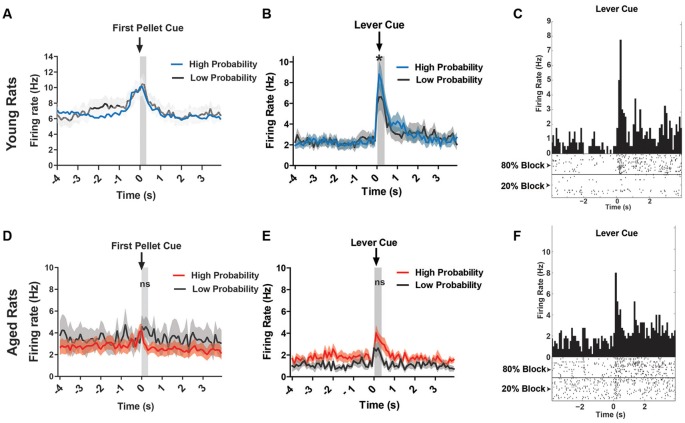
**(A)** Mean firing rate (Hz) of young rats’ VTA neurons (*n* = 151) centered around the first pellet cue (±4.0 s). **(B)** Mean firing rate (Hz) of young rats’ lever excited VTA (DA + nonDA) neurons (*n* = 37) centered around the lever cue (±4.0 s). Blue traces indicate responses during the 80% block; gray traces represent the 20% block. The neurons displayed significantly greater firing rate responses to the lever cue during the high probability block compared to the low probability block (one-tailed dependent *t*-test, *t*_(36)_ = 1.73, *p* = 0.04). **(C)** Peri-event time histogram (PETH) of a lever excited young neuron’s responses to the lever cue during high and low probability blocks. Left Y-axis represents the mean firing rate (Hz) over all the events; time 0 indicates the onset of lever cue. Raster diagrams show individual spikes around the lever cue. Top: 80% block; bottom: 20% block. **(D)** Mean firing rate (Hz) of aged rats’ VTA neurons (*n* = 107) centered around the first pellet cue (±4.0 s). **(E)** Mean firing rate (Hz) of aged rats’ lever excited VTA (DA + nonDA) neurons (*n* = 9) centered around the lever cue (±4.0 s). Red traces indicate responses during the 80% block; gray traces represent the 20% block. The neurons did not display a significant difference in firing rate between the two probability blocks (one-tailed dependent *t*-test, *t*_(8)_ = 0.09, *p* = 0.46). **(F)** PETH of a lever excited aged neuron’s responses to the lever cue during high and low probability blocks. Left Y-axis represents the mean firing rate (Hz) over all the events; time 0 indicates the onset of lever cue. Raster diagrams show individual spikes around the lever cue. Top: 80% block; bottom: 20% block. *p < 0.05; ns = not significant, *p* > 0.05.

Since the DA waveform analysis could exclude DA neurons in the aged rats due to functional changes in the neurons over time, we could be neglecting neurons that do indeed encode the salient events of the task. Thus, the neural data were reanalyzed to include all VTA neurons for both age groups. Previous reports show that even non-DA neurons of the VTA encode salient events (Puryear et al., 2010), albeit with different firing patterns in response to reward-predicting cues (Cohen et al., 2012). When examining all young rats’ VTA neurons response to reward, or first pellet cue, we found that the firing rate was significantly higher during this cue period compared to baseline (two-way ANOVA, significant main effect of time, *F*_(1,568)_ = 10.87, *p* = 0.001; Figure 6A), but there was no significant difference between probability blocks to this cue (no main effect of probability, *F*_(1,568)_ = 0.13, *p* = 0.72; Figure 6A). In the same analysis on aged rats’ VTA neurons, there was no significant difference in firing rate between baseline and the cue, or between probability blocks (two-way ANOVA, no effect of time, *F*_(1,330)_ = 0.33, *p* = 0.56; or probability, *F*_(1,330)_ = 1.83, *p* = 0.18; Figure 6D).

In the present study, for the young rats, 37/154 (24.0%) of neurons displayed a pattern of phasic excitation to the lever cue (Figure 6B). The responses of these neurons during the first 300 ms after the onset of the lever cue were compared between the high and low probability blocks to see if the phasic excitation of these neurons scaled with the probability of receiving the large reward. Indeed, the neurons displayed significantly greater firing rate responses to the lever cue during the high probability block compared to the low probability block (one-tailed dependent *t*-test, *t*_(36)_ = 1.73, *p* = 0.04; Figure 6B). For the aged rats, only 9/115 (7.8%) of neurons showed excitation to the lever cue and when these responses were analyzed to examine if they encoded the probability of available reward it was found that the neurons did not display a difference in firing rate between the two probability blocks (one-tailed dependent *t*-test, *t*_(8)_ = 0.09, *p* = 0.46). Therefore, the aged rats’ VTA neurons did not distinguish between the cues that predict the probabilistic rewards with high and low probabilities while the young rats VTA neurons did. This is evident even in individual neurons’ responses to the lever cue in young and aged rats (Figures 6C,F). These results indicate that the increase in probability discounting in aged rats may be due to the blunting of the dynamic range of the VTA neurons’ responses, which might prevent the aged rats from correctly assessing the value of the risky option.

## Discussion

### Age-Related Increase in Probability Discounting

It is important to understand how and why risk-based decision making changes over the lifespan. The current data show that aged rats display a decreased choice of the large risky reward, i.e., more discounting on the probability discounting task, reflecting an age-related change in risk-based decision making. Previous reports have not found this same age-related effect on probability discounting in rats (Gilbert et al., 2012; Samson et al., 2015). This apparent discrepancy could be due to rat strain differences, i.e., F344 rats in Gilbert et al. (2012) and Samson et al. (2015), or a difference in methodologies rather than a lack of an age-related effect on probability discounting. For example, in Gilbert et al. (2012), the reward was grain-based rather than sucrose and the large reward was twice the size of the small, vs. four times in our current study. These differences in reward palatability and size could affect the computations animals make when considering their options. The findings here are in line with increased risk-aversion observed in older humans (e.g., Lee et al., 2008). Importantly, differences in choice behavior were not due to increased omitted trials for aged rats. Thus, we believe our model of risk-based decision making can be used to study the underlying physiological changes in the brain that contribute to the observed alterations in decision making with age.

### Age-Related Reduction in Response to Positive, Not Negative, Reinforcement

We found that the increased discounting was related to alterations in choices after positive reinforcement as aged rats show decreased win-stay behavior compared to their younger counterparts. It has previously been shown that aged rats have a deficit in repeating a choice that was previously rewarded (Means and Holsten, 1992). On the other hand, we did not find a significant difference between the age groups in lose-shift behavior, indicating that aged animals are not necessarily more loss-averse than their younger counterparts. Therefore, age-related increases in discounting on the probability task are more likely to be due to changes in adequately assessing the positive outcomes of past choices. This effect seems to be selective for risky choices, as the choice of the large/risky reward was not significantly different between aged and young rats when outcomes were certain, i.e., when the probability of the “risky” reward was 100%. Instead, deficits arose when risk was introduced, even when it is more beneficial to continue choosing the risky option, i.e., during the 50% block. This is in line with human research that shows older individuals selectively avoid risky options, even if such choices reduce overall gains (Lee et al., 2008; Rutledge et al., 2016).

### Age-Related Changes in Spatial Memory and Probability Discounting Are Not Strongly Coupled

Age-related decline of hippocampal function has been vigorously studied in rats using spatial memory tasks like the Morris water maze. These tests reveal that aged rats consistently show impairment on hippocampal-dependent tasks compared to young rats (Gallagher et al., 2011). Indeed, *in vivo* investigations into hippocampal function reveal that place cells are less likely to update in new environments, and this decrease in adaptability is correlated with deficits in spatial memory (Wilson et al., 2004). Consistent with this literature, aged rats tested in this study showed significantly impaired water maze performance compared to younger animals. We were interested in whether this decline in spatial memory was correlated with altered risk-based decision making. Although it is thought that cognitive decline with age in one brain region is not necessarily predictive of decline in other regions, most of this research is restricted to assessing the relationship between PFC and hippocampal-dependent tasks (Fiorillo et al., 2003; Moore et al., 2009; Fletcher and Rapp, 2012). We found that choice behavior on the probability discounting task was not significantly related to water maze performance. This is consistent with previous similar studies that assessed whether there is a relationship between the two types of behavioral performances (Gilbert et al., 2012; Samson et al., 2015). Therefore, there is insufficient data at this time to link spatial working memory decline in age with increased probability discounting. With additional study, it would be of interest to determine whether, as has been done with studies of age-related memory changes, further exploitation of individual differences in discounting task performance will reveal new insights into underlying age-related brain mechanisms of decision making (Orsini et al., 2019).

### Age-Related Reduction in VTA Dopamine Responses to Reward

The *in vivo* electrophysiological data recorded from young and aged rats’ VTA show a striking lack of dynamic range in aged VTA neuron responses. This result supports the view that a functional decline in these neurons could ultimately contribute to altered risk-based decision making. The reduced responsiveness of aged VTA neurons to rewarding stimuli may relate to the age-related reduced sensitivity to positive reinforcement observed in the win-stay analysis. Known cellular changes in aged DA neural properties (e.g., Branch et al., 2014) may underlie the low rate and lack of responses by putative DA neurons. For this reason, at this time, we cannot be confident that the neurons classified as dopaminergic in the aged rats are truly dopaminergic due to the possibility that the waveform characteristics may have changed with age (Rollo, 2009; Branch et al., 2014). Similarly, cells classified as non-dopaminergic may indeed be (aged) dopamine neurons. Even so, reduce dopaminergic function in aged rats could contribute to the reduced selection of the risky reward during probability discounting. Previous research indicates that both D1 and D2 dopamine receptor antagonism can reduce the preference for the large risky reward in a similar probability discounting task (St. Onge and Floresco, 2009). Additionally, there is a positive correlation between enhanced DA release in the nucleus accumbens and increased risky decision-making (Freels et al., 2020). It remains to be determined whether the increased risky choice could be accounted for by a change in the impacts of uncertainty on reward value and/or a change in the salience of rewards that drive behaviors (e.g., Roesch et al., 2012). Additionally, future work is needed to confirm the neurochemical identity of recorded VTA neurons of aged rats in order to elucidate if the blunted responses are specifically due to changes in dopamine neurons and their impact on the broader neural circuits within which they operate to affect learning and memory.

There was not a significant difference in behavior between the two age groups in the two-probability block recording task. However, the lack of a striking behavioral effect between the groups in the two-probability block is not surprising given 80% and 20% of four times a reward is a clearly superior and inferior choice, respectively. The behavioral differences between the two age groups are more obvious when the choice is more difficult if one is risk-averse.

## Conclusion

Normal aging is known to affect a wide range of cognitive abilities. The current data support previous research that showed advanced aging affects how decisions are made in risky situations. In particular, we showed that this change may be related to reduced responses to positive, not negative, reinforcement. The latter was supported by additional findings that the aged VTA DA cells are less responsive to rewards when compared to similar cells recorded from young animals. A useful model for examining risky decision-making alteration late into the lifespan can help us understand how older individuals make decisions when faced with uncertain situations. Given the projected increases in the aged population across the world, understanding these changes has important implications for society. Additionally, understanding the physiological changes the brain undergoes with age can aide us in developing treatments to ameliorate any deleterious effects aging may have on cognition.

## Data Availability Statement

All datasets generated for this study are included in the article.

## Ethics Statement

The animal study was reviewed and approved by University of Washington’s and National Institute of Aging’s Institutional Animal Care and Use Committees.

## Author Contributions

VT and SM contributed to the conception and design study. VT, PB, and JL performed the described experiments. VT and JL performed data analysis. VT submitted an earlier draft of the manuscript in partial fulfillment of a Ph.D. dissertation. All authors contributed to the comments and then approved the submitted version.

## Conflict of Interest

The authors declare that the research was conducted in the absence of any commercial or financial relationships that could be construed as a potential conflict of interest.

## References

[B1] Bäckman L. Lindenberger U. Li S. C. Nyberg L. (2010). Linking cognitive aging to alterations in dopamine neurotransmitter functioning: recent data and future avenues. Neurosci. Biobehav. Rev. 34, 670–677. 10.1016/j.neubiorev.2009.12.008 20026186

[B2] Bäckman L. Nyberg L. Lindenberger U. Li S. C. Farde L. (2006). The correlative triad among aging, dopamine, and cognition: current status and future prospects. Neurosci. Biobehav. Rev. 30, 791–807. 10.1016/j.neubiorev.2006.06.005 16901542

[B3] Barense M. D. Fox M. T. Baxter M. G. (2002). Aged rats are impaired on an attentional set-shifting task sensitive to medial frontal cortex damage in young rats. Learn. Mem. 9, 191–201. 10.1101/lm.48602 12177232 PMC182583

[B4] Bizon J. L. LaSarge C. L. Montgomery K. S. McDermott A. N. Setlow B. Griffith W. H. (2009). Spatial reference and working memory across the lifespan of male Fischer 344 rats. Neurobiol. Aging 30, 646–655. 10.1016/j.neurobiolaging.2007.08.004 17889407 PMC2703480

[B5] Branch S. Y. Sharma R. Beckstead M. J. (2014). Aging decreases L-type calcium channel currents and pacemaker firing fidelity in substantia nigra dopamine neurons. J. Neurosci. 34, 9310–9318. 10.1523/jneurosci.4228-13.2014 25009264 PMC4087208

[B300] Cardinal R. N. Howes N. J. (2005). Effects of lesions of the nucleus accumbens core on choice between small certain rewards and large uncertain rewards in rats. BMC Neurosci. 6:37. 10.1186/1471-2202-6-37 15921529 PMC1177958

[B6] Cohen J. Y. Haesler S. Vong L. Lowell B. B. Uchida N. (2012). Neuron-type-specific signals for reward and punishment in the ventral tegmental area. Nature 482, 85–88. 10.1038/nature10754 22258508 PMC3271183

[B7] Cruz-Muros I. Afonso-Oramas D. Abreu P. Barroso-Chinea P. Rodríguez M. González M. C.. (2007). Aging of the rat mesostriatal system: differences between the nigrostriatal and the mesolimbic compartments. Exp. Neurol. 204, 147–161. 10.1016/j.expneurol.2006.10.004 17112516

[B8] Dreher J. C. Meyer-Lindenberg A. Kohn P. Berman K. F. (2008). Age-related changes in midbrain dopaminergic regulation of the human reward system. Proc. Natl. Acad. Sci. U S A 105, 15106–15111. 10.1073/pnas.0802127105 18794529 PMC2567500

[B9] Dumitriu D. Hao J. Hara Y. Kaufmann J. Janssen W. G. Lou W.. (2010). Selective changes in thin spine density and morphology in monkey prefrontal cortex correlate with aging-related cognitive impairment. J. Neurosci. 30, 7507–7515. 10.1523/jneurosci.6410-09.2010 20519525 PMC2892969

[B10] Fields H. L. Hjelmstad G. O. Margolis E. B. Nicola S. M. (2007). Ventral tegmental area neurons in learned appetitive behavior and positive reinforcement. Annu. Rev. Neurosci. 30, 289–316. 10.1146/annurev.neuro.30.051606.094341 17376009

[B11] Fiorillo C. D. Tobler P. N. Schultz W. (2003). Discrete coding of reward probability and uncertainty by dopamine neurons. Science 299, 1898–1902. 10.1126/science.1077349 12649484

[B301] Fletcher B. R. Rapp P. R. (2012). Normal Neurocognitive Aging. Handbook of Psychology. 2nd Edn.

[B12] Freels T. G. Gabriel D. B. Lester D. B. Simon N. W. (2020). Risky decision-making predicts dopamine release dynamics in nucleus accumbens shell. Neuropsychopharmacology 45, 266–275. 10.1038/s41386-019-0527-0 31546248 PMC6901435

[B13] Gallagher M. Burwell R. Burchinal M. R. (1993). Severity of spatial learning impairment in aging: development of a learning index for performance in the Morris water maze. Behav. Neurosci. 107, 618–626. 10.1037/0735-7044.107.4.618 8397866

[B15] Gallagher M. Bizon J. L. Hoyt E. C. Helm K. A. Lund P. K. (2003). Effects of aging on the hippocampal formation in a naturally occurring animal model of mild cognitive impairment. Exp. Gerontol. 38, 71–77. 10.1016/s0531-5565(02)00159-6 12543263

[B14] Gallagher M. Stocker A. M. Koh M. T. (2011). Mindspan: lessons from rat models of neurocognitive aging. ILAR J. 52, 32–40. 10.1093/ilar.52.1.32 21411856 PMC3199952

[B17] Gilbert R. J. Mitchell M. R. Simon N. W. Bañuelos C. Setlow B. Bizon J. L. (2012). Risk, reward, and decision-making in a rodent model of cognitive aging. Front. Neurosci. 5:144. 10.3389/fnins.2011.00144 22319463 PMC3250056

[B18] Goudsmit E. Feenstra M. G. P. Swaab D. F. (1990). Central monoamine metabolism in the male Brown-Norway rat in relation to aging and testosterone. Brain Res. Bull. 25, 755–763. 10.1016/0361-9230(90)90054-4 2289164

[B302] Jin X. Costa R. M. (2010). Start/stop signals emerge in nigrostriatal circuits during sequence learning. Nature 466, 457–462. 10.1038/nature09263 20651684 PMC3477867

[B19] Jo Y. S. Lee J. Mizumori S. J. (2013). Effects of prefrontal cortical inactivation on neural activity in the ventral tegmental area. J. Neurosci. 33, 8159–8171. 10.1523/jneurosci.0118-13.2013 23658156 PMC3675177

[B20] Lee T. M. Leung A. W. Fox P. T. Gao J. H. Chan C. C. (2008). Age-related differences in neural activities during risk taking as revealed by functional MRI. Soc. Cogn. Affect. Neurosci. 3, 7–15. 10.1093/scan/nsm033 19015090 PMC2569821

[B21] Mata R. Josef A. K. Samanez-Larkin G. R. Hertwig R. (2011). Age differences in risky choice: a meta-analysis. Ann. N Y Acad. Sci. 1235, 18–29. 10.1111/j.1749-6632.2011.06200.x 22023565 PMC3332530

[B22] Means L. W. Holsten R. D. (1992). Individual aged rats are impaired on repeated reversal due to loss of different behavioral patterns. Physiol. Behav. 52, 959–963. 10.1016/0031-9384(92)90377-e 1484853

[B23] Moore T. L. Schettler S. P. Killiany R. J. Rosene D. L. Moss M. B. (2009). Effects on executive function following damage to the prefrontal cortex in the rhesus monkey (*Macaca mulatta*). Behav. Neurosci. 123, 231–241. 10.1037/a0014723 19331446

[B24] Morrison J. H. Baxter M. G. (2012). The ageing cortical synapse: hallmarks and implications for cognitive decline. Nat. Rev. Neurosci. 13, 240–250. 10.1038/nrn3200 22395804 PMC3592200

[B25] Morrison J. H. Hof P. R. (1997). Life and death of neurons in the aging brain. Science 278, 412–419. 10.1126/science.278.5337.412 9334292

[B303] Mather M. Mazar N. Gorlick M. A. Lighthall N. R. Burgeno J. Schoeke A.. (2012). Risk preferences and aging: the “certainty effect” in older adults’ decision making. Psychol. Aging 27:801. 10.1037/a0030174 23066800 PMC3565580

[B26] Nicolle M. M. Colombo P. J. Gallagher M. McKinney M. (1999). Metabotropic glutamate receptor-mediated hippocampal phosphoinositide turnover is blunted in spatial learning-impaired aged rats. J. Neurosci. 19, 9604–9610. 10.1523/jneurosci.19-21-09604.1999 10531462 PMC6782926

[B27] Orsini C. A. Hernandez C. M. Bizon J. L. Setlow B. (2019). Deconstructing value-based decision making *via* temporally selective manipulation of neural activity: insights from rodent models. Cogn. Affect. Behav. Neurosci. 19, 459–476. 10.3758/s13415-018-00649-0 30341621 PMC6472996

[B28] Puryear C. B. Kim M. J. Mizumori S. J. (2010). Conjunctive encoding of movement and reward by ventral tegmental area neurons in the freely navigating rodent. Behav. Neurosci. 124, 234–247. 10.1037/a0018865 20364883 PMC2864532

[B30] Rapp P. R. Deroche P. S. Mao Y. Burwell R. D. (2002). Neuron number in the parahippocampal region is preserved in aged rats with spatial learning deficits. Cereb. Cortex 12, 1171–1179. 10.1093/cercor/12.11.1171 12379605

[B29] Rapp P. R. Gallagher M. (1996). Preserved neuron number in the hippocampus of aged rats with spatial learning deficits. Proc. Natl. Acad. Sci. U S A 93, 9926–9930. 10.1073/pnas.93.18.9926 8790433 PMC38531

[B31] Roesch M. R. Calu D. J. Schoenbaum G. (2007). Dopamine neurons encode the better option in rats deciding between differently delayed or sized rewards. Nat. Neurosci. 10, 1615–1624. 10.1038/nn2013 18026098 PMC2562672

[B32] Roesch M. R. Esber G. R. Bryden D. W. Cerri D. H. Haney Z. R. Schoenbaum G. (2012). Normal aging alters learning and attention-related teaching signals in basolateral amygdala. J. Neurosci. 32, 13137–13144. 10.1523/jneurosci.2393-12.2012 22993430 PMC3461330

[B33] Rollo C. D. (2009). Dopamine and aging: intersecting facets. Neurochem. Res. 34, 601–629. 10.1007/s11064-008-9858-7 18841466

[B34] Rosenzweig E. S. Barnes C. A. (2003). Impact of aging on hippocampal function: plasticity, network dynamics, and cognition. Prog. Neurobiol. 69, 143–179. 10.1016/s0301-0082(02)00126-0 12758108

[B35] Rutledge R. B. Smittenaar P. Zeidman P. Brown H. R. Adams R. A. Lindenberger U.. (2016). Risk taking for potential reward decreases across the lifespan. Curr. Biol. 26, 1634–1639. 10.1016/j.cub.2016.05.017 27265392 PMC4920952

[B36] Samanez-Larkin G. R. Gibbs S. E. Khanna K. Nielsen L. Carstensen L. L. Knutson B. (2007). Anticipation of monetary gain but not loss in healthy older adults. Nat. Neurosci. 10, 787–791. 10.1038/nn1894 17468751 PMC2268869

[B37] Samson R. D. Venkatesh A. Lester A. W. Weinstein A. T. Lipa P. Barnes C. A. (2015). Age differences in strategy selection and risk preference during risk-based decision making. Behav. Neurosci. 129, 138–148. 10.1037/bne0000037 25664565 PMC4526244

[B38] Schoenbaum G. Setlow B. Saddoris M. P. Gallagher M. (2006). Encoding changes in orbitofrontal cortex in reversal-impaired aged rats. J. Neurophysiol. 95, 1509–1517. 10.1152/jn.01052.2005 16338994 PMC2430623

[B40] St. Onge J. R. Chiu Y. C. Floresco S. B. (2010). Differential effects of dopaminergic manipulations on risky choice. Psychopharmacology 211, 209–221. 10.1007/s00213-010-1883-y 20495787

[B39] St. Onge J. R. Floresco S. B. (2009). Dopaminergic modulation of risk-based decision making. Neuropsychopharmacology 34, 681–697. 10.1038/npp.2008.121 18668030

[B304] Takahashi Y. K. Roesch M. R. Stalnaker T. A. Haney R. Z. Calu D. J. Taylor A. R.. (2009). The orbitofrontal cortex and ventral tegmental area are necessary for learning from unexpected outcomes. Neuron 62, 269–280. 10.1016/j.neuron.2009.03.005 19409271 PMC2693075

[B305] Takahashi Y. K. Roesch M. R. Wilson R. C. Toreson K. O’Donnell P. Niv Y.. (2011). Expectancy-related changes in firing of dopamine neurons depend on orbitofrontal cortex. Nat. Neurosci. 14, 1590–1597. 10.1038/nn.2957 22037501 PMC3225718

[B41] Wilson I. A. Ikonen S. Gureviciene I. McMahan R. W. Gallagher M. Eichenbaum H.. (2004). Cognitive aging and the hippocampus: how old rats represent new environments. J. Neurosci. 24, 3870–3878. 10.1523/jneurosci.5205-03.2004 15084668 PMC6729357

[B42] Wise R. A. (2004). Dopamine, learning and motivation. Nat. Rev. Neurosci. 5, 483–494. 10.1038/nrn1406 15152198

[B43] Zheng W. Ycu E. A. (2012). A fully automated and highly versatile system for testing multi-cognitive functions and recording neuronal activities in rodents. J. Vis. Exp. 63:e3685. 10.3791/3685 22588124 PMC3529495

